# High Willingness to Participate in Partner Notification among Women Attending Reproductive Health and STI Clinics in Shenzhen, China: A Cross-Sectional Study

**DOI:** 10.3390/ijerph17020386

**Published:** 2020-01-07

**Authors:** Rongxing Weng, Weiye Yu, Fuchang Hong, Chunlai Zhang, Lizhang Wen, Feng Wang, Yiting Luo, Jianbin Ye, Fen Tang, Honglin Wang, Xiangsheng Chen, Yumao Cai

**Affiliations:** 1 Shenzhen Center for Chronic Disease Control, Shenzhen 518020, China; wrx0518@163.com (R.W.); ywy2002@126.com (W.Y.); hfc0755@sina.com (F.H.); szzhchl@163.com (C.Z.); 13602609811@139.com (L.W.); biowangfeng@163.com (F.W.); luoyitingcathy@126.com (Y.L.); yejianbin_gd@126.com (J.Y.); tangfen_123@163.com (F.T.); wanghonglin0612@126.com (H.W.); 2 Peking Union Medical College Institute of Dermatology, Chinese Academy of Medical Science and Peking Union Medical College, Nanjing 210042, China; chenxs@ncstdlc.org; 3 National Center for Sexually Transmitted Disease Control, Nanjing 210042, China

**Keywords:** partner notification, *Chlamydia trachomatis*, willingness, factors

## Abstract

Genital Chlamydia trachomatis (CT) is one of the most common sexually transmitted infections (STI) worldwide. We explored the factors associated with willingness to participate in partner notification (PN) among women attending reproductive health and STI clinics in Shenzhen, China. An anonymous questionnaire was used to collect the sociodemographic characteristics, STI histories, and willingness to participate in routine CT screening and partner notification. In total, 87.31% (*n* = 10,780) of participants were willing to notify their sex partner(s) if they were diagnosed with a CT infection. Willingness to complete PN was significantly associated with: being married, residing in Shenzhen ≥1 year, having completed junior college or higher, not currently reporting STI-related symptoms, willing to have routine CT screening, and having a correct understanding of the health sequelae of CT infection. Nearly all women surveyed at reproductive health and STI clinics in Shenzhen reported willingness to complete PN. Promoting PN in these settings could help detect a large number of additional CT cases. Our findings provide evidence and implications for public health interventions on PN and suggest that targeted interventions are urgently needed for particular subpopulations including those not currently married, with shorter residency, lower education, and less awareness about the dangers of CT infection.

## 1. Introduction

Genital *Chlamydia trachomatis* (CT) is one of the most common sexually transmitted infections (STI) worldwide. The World Health Organization (WHO) indicated a substantial global burden of CT infections in 2016, with the pooled prevalence of 3.8% in women and about 124.3 million cases of CT worldwide in women and men aged 15–49 years of age [1]. Previous studies in China also showed a large burden of CT infections, with a prevalence of 4.1% among women [2] and a higher prevalence (10.1%) among female patients attending sexual and reproductive health clinics [3]. Untreated CT infection in women is associated with several serious sequelae including pelvic inflammatory disease, ectopic pregnancy, chronic pelvic pain, and tubal factor infertility [4]. CT infections may also increase the risk of acquiring and transmitting human immunodeficiency virus (HIV), and around 80% of CT cases are asymptomatic [5,6].

In CT intervention programs and guidelines in several countries, including the United Kingdom [7], Canada [8], the Netherlands [9], and Germany [9], partner notification (PN) is an essential individual and public health strategy to avoid re-infection of a treated index patient and reduce the burden of infections [10,11]. To expand the active screening of CT, a program in China called Shenzhen Gonorrhea and Chlamydia intervention pilot (SGCIP) was launched by the Health and Family Planning Commission of Shenzhen Municipality in 2017. This pilot also included PN in the program process. Many individuals would not know about their infections if they were asymptomatic cases [12], and PN is an effective way to expand CT screening to sex partners. Failure to inform sex partners could represent a missed opportunity in the detection and treatment of numerous asymptomatic individuals. A previous study in the United States showed that effective PN could have a major impact on chlamydia prevalence, with millions of infections averted because of partner notification [13]. PN has also been proven to be cost-effective in terms of reducing the HIV burden [14]. The guidelines on HIV self-testing and partner notification from WHO indicated that PN is cost-effective in reaching high-risk individuals with rare harm or violence, and thus providing a strong recommendation for implementing PN [15]. These guidelines also summarized that high proportions of HIV-positive people could be diagnosed through assisted PN services [15]. There are four options for partner notification including provider referral, patient-based partner referral, expedited partner therapy, and contract referral [16], and one infected patient may use different strategies to notify different sex partners. The option of having health department professionals notify the partner(s) of their risk of being infected is considered as provider referral [17]. When index patients inform their partner(s) by themselves, this option is considered as patient-based partner referral [17]. Expedited partner therapy is a partner treatment approach in which the partner(s) of index patients get treatment prior to clinical evaluation [17]. In the contract referral, index patients prefer to inform their partner(s) within a certain time frame and agree that if they do not notify them within this time frame, disease intervention specialists will inform the partner(s) [17].

The rate of PN varies in different studies: 53% successful direct patient referral in sub-Saharan Africa [11], 73% successful PN in Sweden [18], 76.4% successful PN in the United States [19], and 51% successful PN by patient referral card in Peru [20]. Also, improving the efficacy of PN was found to be more cost-effective than increasing the coverage of CT screening [10]. From a systematic review, factors of partner notification were reported, such as female, higher education level, and regular partner as facilitators, and being married, stigma, fear of adverse reaction, and risk of intimate partner violence as barriers [11].

However, little is known about the acceptability of PN in the Chinese context and many patients in China may be very sensitive to PN because of social stigma and the fear of relationship breakdown [21]. Understanding what characteristics are associated with the willingness to participate in PN could inform future public health intervention efforts to implement and promote this prevention strategy. This study aimed to explore women’s willingness to participate in PN and identify associated factors among women attending reproductive health and STI clinics in Shenzhen.

## 2. Materials and Methods

### 2.1. Study Setting and Population

Study sites were selected using a stratified purposeful sampling design. Six administrative districts in Shenzhen were included and within the selected districts, four hospitals with a high number of reported CT cases in each district were included for recruitment, except one district with only two hospitals. A total of 22 hospitals were included in the study. Patient eligibility criteria were as follows: (1) cis-gender women, (2) aged 18 to 49, inclusive, (3) having ever engaged in sexual activity, and (4) having not used any antibiotics in the last two weeks. A convenient sampling was used to recruit participants. From 1 April to 16 May 2018, the first 15 eligible patients per day in the obstetrics and gynecology, urology, and dermatology clinics were recruited for the questionnaire survey.

### 2.2. Questionnaire Survey

We provided intensive training in conducting questionnaire surveys for all study staff. The paper-based questionnaire surveys were administered one-on-one by the study staff. A set of anonymous structured questions was used to collect the information about sociodemographic characteristics and STI history (e.g., age, marital status, and chlamydia testing and diagnosis history), willingness to undergo routine CT screening, and willingness to participate in PN.

### 2.3. Questions about CT-Related Knowledge and Willingness to Participate in Partner Notification (PN)

Two questions assessed CT-related knowledge. Knowledge question 1, “What do you know about genital CT infections?” included four response options: (a) “Never heard of it,” (b) “A kind of infectious disease,” (c) “A kind of genital tract infection,” and (d) “A kind of sexually transmitted disease.” Response options were coded as follows: (a) “Lack of understanding,” and (b), (c), or (d) as “Correct understanding,” resulting in two knowledge levels. Knowledge question 2, “What do you know about the dangers of genital *Chlamydia trachomatis* infections for the human body?” included four response options: (a) “No danger,” (b) “May affect sexual life,” (c) “May affect fertility,” and (d) “Know nothing about it.” Response options were coded as follows: (a) “Incorrect understanding,” (b) or (c) “Correct understanding,” and (d) “Lack of understanding,” resulting in three levels of knowledge. The question “If you were diagnosed with CT, would you be willing to inform your partner?” was intended to obtain information on willingness to participate in PN from participants, with two possible responses (“No” and “Yes”).

### 2.4. Statistical Analyses

All questionnaire data were double entered using Epi Data software (Epi Data for Windows; The Epi Data Association, Odense, Denmark). The frequency (%) and mean ± SD (standard deviation) were calculated for categorical variables and continuous variables, respectively. Univariate logistic regression analysis was used to obtain crude odds ratios (OR) and their 95% confidence intervals (CIs). Using a forward stepwise procedure, multivariate logistic regression analysis, including those variables with *p* < 0.10 in the univariate logistic regression analysis, was used to obtain adjusted odds ratios (AOR) and their 95% CIs. We adopted a multivariable logistic regression model, defining the willingness to participate in partner notification as a dependent variable and age groups, marital status, separation, residency, residence time, education level, monthly income, insurance, sexual orientation, STI-related symptoms, willingness to undergo routine CT screening, having a new sexual partner or multiple sex partners in the past three months, and CT knowledge questions 1 and 2 as independent variables. All data analyses were performed with Statistical Package for Social Sciences (SPSS) version 21.0 software (SPSS Inc., Chicago, IL, USA). All tests were two-tailed, and *p* < 0.05 was defined as statistically significant.

### 2.5. Ethical Approval

All participants provided written informed consent. This study was approved by the Ethical Review Committee of the Shenzhen Center for Chronic Disease Control (Approval No. 20180206).

## 3. Results

### 3.1. Sociodemographic Characteristics and Sexually Transmitted Infections (STI) Histories and the Correlates with Willingness to Participate in Partner Notification

The sociodemographic characteristics and STI histories of participants are shown in Table 1. We excluded 483 participants because of incomplete questionnaires, leaving 10,780 participants in the final analysis. The mean age was 31.58 ± 7.09. The majority of participants (85.38%) were over 24 years old and 78.98% were married. One-tenth (10.38%) of the participants lived alone or apart from their husbands or boyfriends, and 29.59% were Shenzhen residents. The education level of two-fifths (40.73%) of the participants was junior college or higher, and 64.38% had health insurance.

Almost all (91.36%) participants had not been tested for CT infections before, and 83.39% had not been diagnosed with a CT infection before. More than half (61.83%) of the participants had STI-related symptoms on the day they completed the questionnaire, and 29.53% of participants had a new sexual partner or multiple sex partners in the last three months.

The correlates with willingness to participate in PN are shown in Table 1. Participants aged >24 were more willing to participate in PN than those aged ≤24. Participants currently married were more willing to participate in PN than those with other marital status. Participants with Shenzhen residency were more willing to participate in PN than those with residency in other cities or provinces. Participants with ≥1 year of residency were more willing to participate in PN than those with <1 year of residency. Higher willingness to participate in PN was also found in participants with a higher education level, higher monthly income, and health insurance; those without a new sexual partner or multiple sex partners; those willing to have routine CT screening; and those with a correct understanding of the sequalae of CT infection.

### 3.2. CT-Related Knowledge and Willingness to Undergo Routine CT Screening and Partner Notification

CT-related knowledge and willingness to undergo routine CT screening and partner notification are presented in Table 1. Almost all participants (90.10%) were willing to have routine CT screening and 87.31% would be willing to notify their sex partners if they were diagnosed with a CT infection. Around three-quarters of respondents lacked understanding of chlamydia infections (knowledge Q1 = 75.30%; Q2 = 72.60%). 

### 3.3. Factors Associated with Willingness to Participate in Partner Notification

The results of both univariate and multivariate logistic regression analysis are shown in Table 2. A total of 13 variables associated with the willingness to participate in partner notification at *p* < 0.10 in the univariate analysis were included in the multivariate logistic regression analysis. The results from the multivariable logistic regression model indicated that the willingness to participate in partner notification was positively associated with being married (OR = 1.53, 95% CI = 1.17–2.01), residing in Shenzhen for one year or more (OR = 2.26, 95% CI = 1.84–2.77), junior college education or higher (OR = 1.61, 95% CI = 1.31–1.99), not currently experiencing STI-related symptoms (OR = 2.01, 95% CI = 1.67–2.42), and being willing to have routine CT screening (OR = 11.75, 95% CI = 9.76–14.14); and negatively associated with having incorrect understanding of the sequalae of CT infection (OR = 0.26, 95% CI = 0.18–0.39).

## 4. Discussion

Our study indicated that the willingness to participate in partner notification in CT infections was very high (87%) among women attending reproductive health and STI clinics in Shenzhen, China. Several studies in different countries have reported similar results. A study in Botswana suggested that most pregnant women (98%) were willing to notify their partner about their STI results [22]. Buchsbaum et al. found that 98% of female adolescents expressed willingness to inform their partner of a STI diagnosis in the southern United States [19]. Results from a systematic review showed that many index patients, ranging from 58 to 93%, were willing to give their STI results to their partner [23]. Our findings provide data in support of regular CT screening projects and suggest that the implementation and promotion of PN in clinics may be helpful for expanding CT screening among high-risk groups. Among the different types of PN, although provider referral has been found to be the most effective strategy to decrease re-infection rates and increase the number of partners treated [24], it may be impractical to manage sex partner notification through provider referral because of the large number of CT cases and limited public health resources. This leaves patient-based referral (the focus of the current study) as the most common and feasible referral strategy for CT infection [25,26]. Even with a relatively lower efficiency than provider referral, patient referral could still help detect a significant number of additional CT cases at low costs.

In our study, married participants were more willing to notify their partner than those with other marital status, which was consistent with the results of two other studies [27,28]. It is suggested that interdependency may be a motivation for married individuals to have a higher concern for their spouse’s health [27], which leads to higher willingness to participate in PN. Our study also found that participants who had lived in Shenzhen longer and who had higher education were more willing to notify their partner about CT infections. Taken together, these findings may be partly explained by Shenzhen’s large floating population—a common phenomenon in China’s modern cities. Migrants in this population tend to be unmarried, have low education, and stay in the city for a short time [29]. The implications of this for successful PN suggest the importance of education-level-appropriate programs on sexual and reproductive health that highlight the health benefits of informing partners.

Our study may be the first survey to report the following findings. Lower willingness was found among participants with STI-related symptoms than those without symptoms. Patients would think that they are closer to the STIs when they have STI-related symptoms, which was proven by a previous study in which around 50% of men said that they would be concerned about chlamydia only if they had symptoms [30]. Because of the self-perceived high risk of STI acquisition, patients with STI-related symptoms may be more concerned about the outcomes of PN such as relationship breakdown and partner violence, and be more cautious about expressing their willingness to engage in PN. However, it was found that there was no association between symptoms and CT infection in many studies [19,31,32,33,34], which means that asymptomatic patients can also be considered to have the same risk of CT infection as symptomatic patients. Because of the low level of awareness about CT infection (Knowledge Q1) in our study, asymptomatic individuals may underestimate their risk of CT infection, and may become cautious about participating in PN after they get diagnosed. A study showed that the rate of successful PN was lower than the rate of willingness [23]. Based on the above findings, targeted interventions should be implemented in symptomatic patients to motivate them to participate in PN, and CT-related information should also be distributed to all patients. Participants who were open to routine CT screening had an 11-fold higher willingness to undergo partner notification. This finding might be explained by a higher concern for one’s health. Those who are willing to have routine CT screening are more likely to be concerned about their health, and thus also willing to notify their partner(s) of a diagnosis. Also, CT screening and PN could be implemented in the same intervention and it has been proven that a combination of CT screening and PN could have the greatest impact on CT prevention [13]. Therefore, a comprehensive intervention focused on the motivations of both PN willingness and CT screening willingness could be further considered in future projects.

An important finding of this study was that participants who incorrectly thought there were no health dangers to CT infection were less willing to inform their partner about CT test results, suggesting that lower health literacy may be an important barrier to PN. Poor knowledge of the sequalae of CT infection could hinder the effectiveness of CT-related interventions such as screening [30], which could also explain the unwillingness to engage in PN in our study. Also, poor knowledge could contribute to an increase in risky behavior and thus increase the risk of STDs/HIV acquisition [35], and poor knowledge could also lead to delays in appropriate treatment [36]. Poor knowledge of STDs was seen in many studies [36,37,38,39] and is considered a major issue in disease prevention, so CT-related education, including information about complications, modes of transmission, and asymptomatic presentation, should be implemented in clinics to increase patient awareness and potentially facilitate PN.

This study has several limitations. First, the convenience sampling method may undermine the representativeness of the sample. This methodological limitation is balanced by the large sample size across six city districts and 22 hospitals. Second, the response rate could not be obtained in our study because related data were not collected. Third, these findings may not be generalizable to other Chinese cities. However, as Shenzhen City is comprised of a majority migrant population, the views represented in our data included those of individuals from many parts of China. Fourth, the social desirability bias related to sexual behavior and health behaviors may have influenced participants’ survey responses. Lastly, our study measured the willingness, not behavioral intentions or actual behavior. Future studies are needed to understand how to translate the high willingness of PN into actual notification or partner treatment. The associations we found (e.g., education level, symptom status) may provide a starting point for developing promising intervention strategies in this area.

## 5. Conclusions

To our knowledge, this is the first large-scale population-based study in China to report the willingness to participate in PN and its associated factors. This study reported a high willingness to participate in PN of CT infections among women attending reproductive health and STI clinics in Shenzhen, and provided evidence for health authorities in China to develop and implement guidelines or interventions for PN in CT infections. Our findings provide evidence and implications for public health interventions on PN and suggest that targeted interventions are urgently needed for particular subpopulations, including those not currently married and those with a shorter residency time, lower education, and less awareness about the dangers of CT infection.

## Figures and Tables

**Table 1:** Sociodemographic characteristics and STI histories of participants and the correlates with willingness to participate in partner notification.

Variables	Total *N* (%) ^a^	Willing to Participate in Partner Notification	Unwilling to Participate in Partner Notification	*χ*^2^ Value	*p* Value
Age groups (*n* = 10,716)				32.078	0.000 **
≤24	1567 (14.62%)	1299 (82.90%)	268 (17.10%)		
>24	9149 (85.38%)	8056 (88.05%)	1093 (11.95%)		
Marital status (*n* = 10,716)				47.042	0.000 **
Single/Divorced/Widowed	2253 (21.02%)	1871 (83.04%)	382 (16.96%)		
Married	8463 (78.98%)	7486 (88.46%)	977 (11.54%)		
Living alone or apart (*n* = 9066)				2.940	0.09
Yes	941 (10.38%)	812 (86.29%)	129 (13.71%)		
No	8125 (89.62%)	7167 (88.21%)	958 (11.79%)		
Shenzhen resident (*n* = 10,570)				58.501	0.000 **
No	7442 (70.41%)	6391 (85.88%)	1051 (14.12%)		
Yes	3128 (29.59%)	2855 (91.27%)	273 (8.73%)		
Length of residency (*n* = 10,616)				150.454	0.000 **
<1 year	1366 (12.87%)	1052 (77.01%)	314 (22.99%)		
≥1 year	9250 (87.13%)	8218 (88.84%)	1032 (11.16%)		
Education level (*n* = 10,674)				84.705	0.000 **
High school or lower	6327 (59.27%)	5376 (84.97%)	951 (15.03%)		
Junior college or higher	4347 (40.73%)	3955 (90.98%)	392 (9.02%)		
Monthly income (RMB) (*n* = 9838)				39.969	0.000 **
0–4999	4213 (42.82%)	3608 (85.64%)	605 (14.36%)		
5000–9999	4101 (41.69%)	3581 (87.32%)	520 (12.68%)		
10,000+	1524 (15.49%)	1401 (91.93%)	123 (8.07%)		
Health insurance (*n* = 10,655)				25.731	0.000 **
No	3795 (35.62%)	3230 (85.11%)	565 (14.89%)		
Yes	6860 (64.38%)	6073 (88.53%)	787 (11.47%)		
Sexual orientation (*n* = 10,573)				3.830	0.05
Homosexuality/bisexuality	150 (1.42%)	139 (92.67%)	11 (7.33%)		
Heterosexuality	10423 (98.58%)	9102 (87.33%)	1321 (12.67%)		
Ever CT tested (*n* = 10,517)				1.692	0.19
No	9608 (91.36%)	8386 (87.28%)	1222 (12.72%)		
Yes	909 (8.64%)	807 (88.78%)	102 (11.22%)		
Ever CT diagnosed (*n* = 10,669)					
No	8897 (83.39%)	7764 (87.27%)	1133 (12.73%)	1.475	0.48
Yes	642 (6.02%)	569 (88.63%)	73 (11.37%)		
Forgot	1130 (10.59%)	979 (86.64%)	151 (13.36%)		
Current STI-related symptoms (*n* = 10,663)				130.905	0.000 **
Yes	6593 (61.83%)	5562 (84.36%)	1031 (15.64%)		
No	4070 (38.17%)	3743 (91.97%)	327 (8.03%)		
Having a new sexual partner or multiple sex partners, past 3 months (*n* = 10,628)				3.879	0.04 *
Yes	3138 (29.53%)	2711 (86.39%)	427 (13.61%)		
No	7490 (70.47%)	6575 (87.78%)	915 (12.22%)		
CT screening willingness (*n* = 10,366)				1774.269	0.000 **
Unwilling	1026 (9.90%)	467 (45.52%)	559 (54.48%)		
Willing	9340 (90.10%)	8573 (91.79%)	767 (8.21%)		
Knowledge Q1 (*n* = 10,669)				0.952	0.33
Lack of understanding	8034 (75.30%)	7006 (87.20%)	1028 (12.80%)		
Correct understanding	2635 (24.70%)	2317 (87.93%)	318 (12.07%)		
Knowledge Q2 (*n* = 10,747)				373.406	0.000 **
Incorrect understanding	296 (2.75%)	151 (51.01%)	145 (48.99%)		
Lack of understanding	7802 (72.60%)	6845 (87.73%)	957 (12.27%)		
Correct understanding	2649 (24.65%)	2389 (90.18%)	260 (9.82%)		
Willingness to participate in partner notification (*n* = 10,780)				-	-
Unwilling	1368 (12.69%)	-	-		
Willing	9412 (87.31%)	-	-		

^a^ %: Constituent ratio. * *p* < 0.05, ** *p* < 0.001. Abbreviations: CT, chlamydia trachomatis; STI, sexually transmitted infections.

**Table 2:** Logistic regression analysis of factors associated with willingness to participate in partner notification.

Variables	Crude OR (95% CI)	*p* Values	Adjusted OR (95% CI)	*p* Values
Age groups				
≤24	Reference		Reference	
>24	1.52 (1.31,1.76)	0.000 **	1.12 (0.85,1.48)	0.420
Marital status				
Single/Divorced/Widowed	Reference		Reference	
Married	1.56 (1.38,1.78)	0.000 **	1.53 (1.17,2.01)	0.002 *
Living alone or apart				
Yes	Reference		Reference	
No	1.19 (0.98,1.45)	0.087 *	1.01 (0.79,1.31)	0.913
Shenzhen resident				
No	Reference		Reference	
Yes	1.72 (1.49,1.98)	0.000 **	0.97 (0.79,1.21)	0.809
Length of residency				
<1 year	Reference		Reference	
≥1 year	2.38 (2.06,2.74)	0.000 **	2.26 (1.84,2.77)	0.000 **
Education level				
High school or lower	Reference		Reference	
Junior college or higher	1.78 (1.58,2.02)	0.000 **	1.61 (1.31,1.99)	0.000 **
Monthly income (RMB)				
0–4999	Reference		Reference	
5000–9999	1.15 (1.02,1.31)	0.025 *	1.00 (0.83,1.20)	0.963
10,000+	1.91 (1.56,2.34)	0.000 **	1.20 (0.89,1.62)	0.226
Health insurance				
No	Reference		Reference	
Yes	1.35 (1.20,1.52)	0.000 **	1.02 (0.85,1.22)	0.829
Sexual orientation				
Homosexuality/bisexuality	Reference		Reference	
Heterosexuality	0.55 (0.29,1.01)	0.054	0.74 (0.34,1.63)	0.462
Ever CT tested				
No	Reference			
Yes	1.15 (0.93,1.43)	0.194		
Ever CT diagnosed				
No	Reference			
Yes	1.14 (0.88,1.46)	0.316		
Forgot	0.95 (0.79,1.14)	0.552		
Current STI-related symptoms				
Yes	Reference		Reference	
No	2.12 (1.86,2.42)	0.000 **	2.01 (1.67,2.42)	0.000 **
Having a new sexual partner or multiple sex partners, past 3 months				
Yes	Reference		Reference	
No	1.13 (1.00,1.28)	0.049 *	1.09 (0.91,1.31)	0.356
CT Screening willingness				
Unwilling	Reference		Reference	
Willing	13.38 (11.59,15.44)	0.000 **	11.75 (9.76,14.14)	0.000 **
Knowledge Q1				
Correct understanding	Reference			
Lack of understanding	0.94 (0.82,1.07)	0.329		
Knowledge Q2				
Correct understanding	Reference		Reference	
Lack of understanding	0.78 (0.67,0.90)	0.001 *	0.91 (0.74,1.11)	0.342
Incorrect understanding	0.11 (0.09,0.15)	0.000 **	0.26 (0.18,0.39)	0.000 **

* *p* < 0.05, ** *p* < 0.001. Abbreviations: OR, odds ratio; CI, confidence interval; CT, chlamydia trachomatis; STI, sexually transmitted infections.
